# Compressive and Flexural Strengths of EVA-Modified Mortars for 3D Additive Construction

**DOI:** 10.3390/ma12162600

**Published:** 2019-08-15

**Authors:** Kyu-Seok Yeon, Kwan Kyu Kim, Jaeheum Yeon, Hee Jun Lee

**Affiliations:** 1 Department of Regional Infrastructure Engineering, Kangwon National University, Chuncheon, Gangwon 24341, Korea; 2 North Gyeonggi Branch, Joongbu Division, Korea Conformity Laboratories, Pocheon, Gyeonggi 11184, Korea; 3 Department of Engineering and Technology, Texas A&M University-Commerce, Commerce, TX 75429, USA

**Keywords:** EVA-modified mortars, compressive strength, flexural strength, curing age, EVA/cement ratio, cast specimens, printed specimens

## Abstract

The compressive and flexural strengths of mortars modified with ethylene-vinyl acetate (EVA) were experimentally investigated for use in three-dimensional (3D) additive construction (3DAC). EVA powder, which is available in a premix type, was employed as an admixture. The test results for the cast specimens showed that, at a curing age of 28 days, the compressive strengths ranged from 32.92 MPa to 43.50 MPa, and the flexural strengths ranged from 12.73 MPa to 14.49 MPa. The compressive and flexural strengths of the printed specimens were relatively lower: 23% to 26% and 3% to 7%, respectively. The compressive strength also decreased and the flexural strength increased when the EVA/cement ratio was increased. The results of the experiment reveal that the EVA-modified mortar had a high rate of strength development early on, making the material advantageous for use in 3DAC. It was determined that the appropriate EVA/cement ratio ranged between 5% and 15%. However, the printed specimens exhibited lower compressive and flexural strengths than did the cast specimens, and the compressive strength decreased as the EVA content was increased. This study provides the compressive and flexural strengths of common EVA-modified mortars, important data for 3DAC applications.

## 1. Introduction

Research is being actively carried out in various industrial fields in an effort to develop new technologies to enhance productivity. Among these technologies, three-dimensional (3D) printing allows the user to produce desired products without fabricating each individual part or engaging in a repetitious assembly process. Even geometrically complex shapes can be fashioned with precision [1]. Such 3D printing technology represents a paradigm shift from a conventional labor-intensive industry to one of automated production. This new manufacturing technology was actively adopted in various fields and is widely being used [2].

In particular, the manufacturing, medical, and foods industries are actively engaged in research on ways of utilizing 3D printers [3,4,5]. In 1997, Pegna [6] became the first to apply 3D printing technology to a construction project. After Khoshnevis [7], a professor at the University of Southern California, introduced contour crafting (the current form of 3D additive construction, commonly referred to as 3DAC), 3D printing technology garnered increasing interest in the construction industry.

The 3DAC technique consists of a concrete printer, 3D modeling software, and the material used in printing. These three components can be independently considered. The fields of specialization related to these components include mechanical elements, 3D modeling using computers, and the cementitious materials employed in printing [8]. Among these three research areas, cementitious printing materials are key to 3DAC, and their importance was highlighted accordingly. A new committee (i.e., Committee 564: 3D Printing with Cementitious Materials) focusing on cementitious materials for 3D printing was organized by the American Concrete Institute (ACI) in 2018, commencing activities in March 2019 [9]. Thus, research regarding cement-based 3DAC materials is expected to become increasingly common. However, additional work is required to establish appropriate standards for the 3DAC process. Currently, there are no specifications of printing, such as the acceptable range of cross-section sizes for the extrusion nozzle, layer height, printing speed, layer cycle-time, etc. [10].

Recent work on concrete materials used in 3DAC mainly employed silica fume, fly ash, superplasticizers, and viscosity-modifying agents as admixture in the cement [11,12]. Numerous studies were also conducted on the mix proportions and properties of mortars modified with ethylene-vinyl acetate (EVA). However, the results of these existing EVA-modified mortars cannot be applied to the 3DAC process because low workability is required; 3DAC materials must be added layer upon layer after extrusion. If EVA powder is employed as an admixture in cement mortar or concrete, the workability is improved. This is due to the ball-bearing action of the dispersible EVA powder [13]. Also, adhesion at the interface between the EVA-modified mortar and attached materials is improved due to the polymer film produced by the EVA powder [14,15]. EVA also offers improved water resistance and high resistance to the diffusion of chloride ions, oxygen, and carbon dioxide [16]. Yet another important feature is that EVA-modified mortars can be employed as a pre-mixed 3DAC material; dry mix is available with EVA powder, cement, aggregates, etc. [13]. Thus, the use of EVA-modified mortars can offer a notable advantage in 3DAC because they provide a reduction in both the manpower and cost associated with handling and transporting materials. It is this fact that motivates the present study.

The requirements for proper use of 3DAC material can be broken down into two categories: the material’s fresh and hardened properties. The fresh properties required include flowability, extrudability, buildability, and open time. The necessary hardened properties consist of compressive and flexural strengths [17,18,19]. The fresh properties of EVA-modified mortars were studied by Yeon et al. [20], and the optimal flow of EVA-modified mortars was determined. As a next step, this study experimentally investigated the strength development of EVA-modified mortars, in order to determine whether such materials would be feasible for 3DAC use. This work is significant because the compressive and flexural strengths of EVA-modified mortars, which are crucial to determining the stability of a structure, were investigated via two types of specimens that were produced for comparison purposes (i.e., cast and printed).

## 2. Experiment Program

### 2.1. Specimen Preparation

#### 2.1.1. Cast Specimen

The cast specimens were produced in accordance with ASTM C348-14: Standard Test Method for Flexural Strength of Hydraulic-Cement Mortars [21]. However, the standard flow of 110 ± 5% proposed in ASTM C348-14 [21] was not applied when producing the cast specimens (40 × 40 × 160 mm). Instead, these specimens were produced based on a flow of 65% [20], which is optimal when using EVA-modified mortars for 3DAC. All cast specimens were cured for specific aging days (i.e., 1, 3, 7, and 28 days) in a constant temperature and humidity chamber set to a temperature of 23 ± 2 °C and humidity of 65% ± 5%. The specimens were demolded after 24 h.

#### 2.1.2. Printed Specimen 

**Figure 1:**
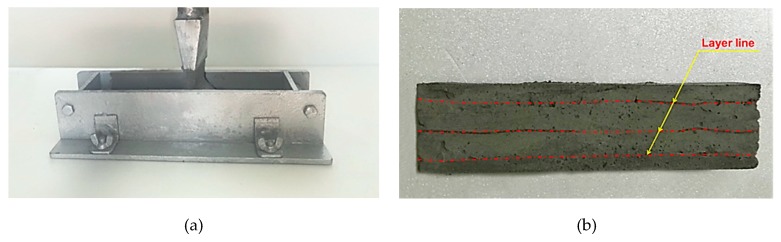
Preparation of the printed specimens: (**a**) Specimen production using 3DAC process; (**b**) Produced specimen using 3DAC process.

Since the configuration of the printed specimen could not be satisfied precisely with respect to the standard specimen configuration (40 × 40 × 160 mm) proposed in ASTM C348-14 [21], the formwork was used as a guide for the layers of EVA-modified mortar being stacked (see Figure 1). The printed specimens (40 × 40 × 160 mm) were produced by directly printing the EVA-modified mortar into a mold, without any extra vibration or compaction. After demolding the formwork, the surface of the printed specimen was pressed very lightly with a trowel to smooth the surface. It took about 30–35 s to print out one layer. However, the speed of the extrusion was not considered to be an experimental parameter in this study. After casting, the mixtures were cured for specific numbers of aging days (i.e., 1, 3, 7, and 28 days) in curing conditions identical to those of the cast specimens. The specimens were demolded after 24 h, as were the cast specimens. The custom-made extrusion-based concrete printer used to print the EVA-modified mortars consisted of a peristaltic pump (i.e., squeeze pump) and nozzle with a cross-section of 36 × 10 mm.

### 2.2. Materials and Mix Proportions

#### 2.2.1. Materials

The materials used in this study were ordinary Portland cement (Type 1), silica sand, fly ash, silica fume, a superplasticizer, a viscosity-modifying agent, and EVA powder. The properties of these materials are shown Table 1, Table 2, Table 3, Table 4, Table 5, Table 6 and Table 7.

#### 2.2.2. Mix Proportions

The optimal flow (i.e., 65%) for EVA-modified mortars that satisfy the fresh property requirements (i.e., flowability, extrudability, buildability, and open time) was determined by Yeon et al. [20] through trial and error. This flow rate of 65% is lower than the standard flow rate of 110% ± 5% proposed by ASTM C109/C109M-02: Testing Method for Compressive Strength of Hydraulic Cement Mortar [22]. Table 8 shows the mix proportions for EVA-modified mortars, as determined based on an optimal flow of 65%. Also, three specimens were tested per these data.

Cement, silica sand, fly ash, silica fume, and EVA powder were dry-mixed. Then, a superplasticizer and viscosity-modifying agent were mixed with water to produce a mixed liquid. The dry-mixed materials and mixed liquid were then poured into a pan mixer for mixing. The mixing procedure was kept the same for both the cast and printed specimens. The expected theoretical density of the EVA-modified mortars that was determined based on the mix proportion provided in Table 8 ranged from 295 kg/cm^3^ to 2364 kg/cm^3^.

### 2.3. Test Method

#### 2.3.1. Compressive Strength

Compressive strength tests were carried out in accordance with ASTM C349-18: Standard Test Method for Compressive Strength of Hydraulic Cement Mortars [23]. The load was applied in a vertical direction from the longitudinal direction of the specimen. The compressive strength was calculated using Equation (1). A universal testing machine (i.e., INSTRON 8502, INSTRON, Norwood, MA, USA) was used for the compressive strength tests.

*f_c_* = *P*/*A*, (1)

where *f_c_* is the compressive strength (MPa), *P* is the maximum load applied to the specimen (N), and *A* is the cross-sectional area of the specimen (mm^2^).

#### 2.3.2. Flexural Strength

Flexural strength tests were carried out in accordance with ASTM C348-14: Standard Test Method for Flexural Strength of Hydraulic Cement Mortars [21]. The same universal testing machine employed in the compressive strength tests was also used to determine flexural strength. The flexural strength tests employed a three-point test method and were calculated using Equation (2).

*f_b_* = 6*M*/*bd*^2^, (2)

where *f_b_* is the flexural strength (MPa), *M* is the maximum bending moment (N·mm), *b* is the width of the specimen (mm), and *d* is the depth of the specimen (mm).

## 3. Results and Discussion

### 3.1. Compressive Strength

#### 3.1.1. Compressive Strengths of Cast Specimens

**Figure 2:**
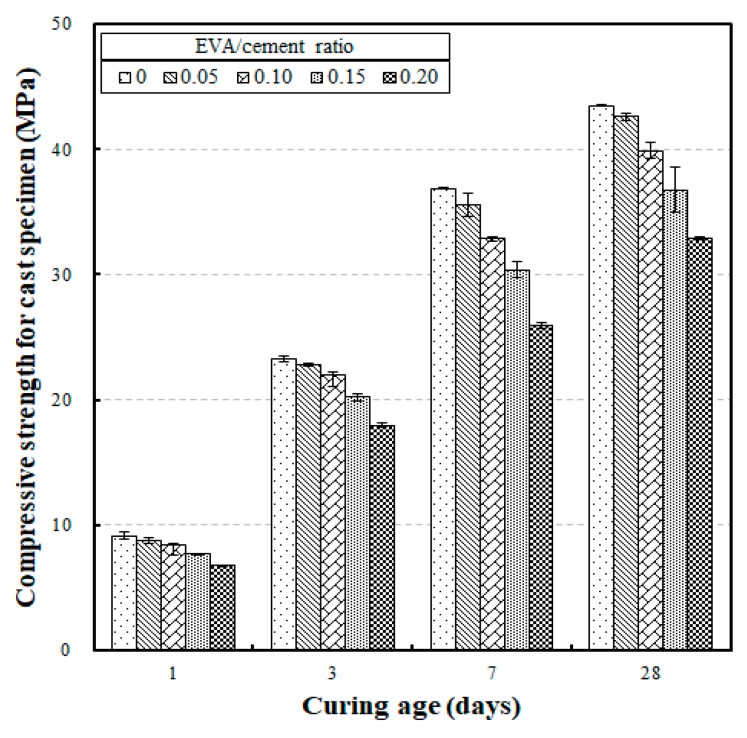
Compressive strength for cast specimens versus curing age according to different ethylene-vinyl acetate (EVA)/cement ratios.

Figure 2 shows the results of the compressive strength tests for the cast specimens according to curing age. The results show that the developed compressive strengths were 6.75 MPa to 9.15 MPa, 17.97 MPa to 23.26 MPa, 25.94 MPa to 36.89 MPa, and 32.92 MPa to 43.50 MPa for curing ages of 1, 3, 7, and 28 days, respectively. These results show an increasing trend in compressive strength as the curing age was increased. Two well-marked properties of EVA-modified mortars were determined through these compressive strength tests. The first was that the strength difference at each curing age (according to the different EVA/cement ratios) significantly increased after seven curing days. The strength differences at curing ages of 1, 3, 7, and 28 days were 2.4 MPa, 5.29 MPa, 10.95 MPa, and 10.58 MPa, respectively. The second standout property was that the rate of strength development at an early curing age was high at the compressive strength at 28 curing days, which was set as the reference (i.e., 100%). Thus, compared to the compressive strength at 28 curing days, the compressive strengths developed from 20.5% to 21.0% at one curing day, 53.4% to 54.6% at three curing days, and 78.8% to 84.8% at seven curing days.

From the results of the strength development tests (as compared to the compressive strength developed at a curing age of 28 days), it was determined that the strengths of the EVA-modified mortars developed at an early age were relatively higher than what was developed by ordinary cement concrete at an early age.

**Figure 3:**
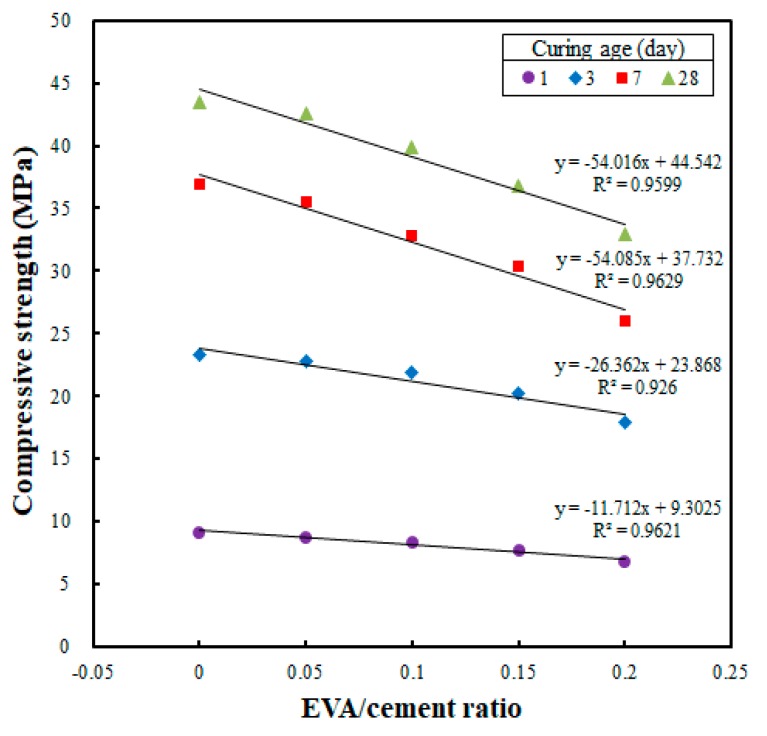
Relationship between EVA/cement ratio and compressive strength for cast specimens at different curing ages.

Figure 3 shows the results of the correlation analysis between the EVA/cement ratio and compressive strength for each curing age; these were collected in order to determine whether the compressive strengths were affected when the EVA/cement ratios were changed. Figure 3 shows that the compressive strength decreased when the EVA/cement ratio was increased. Also, the slope of the linear regression demonstrates that the compressive strength decreased significantly when the curing age was increased. Specifically, the effect of the EVA/cement ratio on compressive strength was examined for cast specimens with a curing age of 28 days (see Figure 3). When the EVA/cement ratio of zero was set as the reference (i.e., 100%), the compressive strength test results showed 97.9%, 91.7%, 84.5%, and 75.6% for the EVA/cement ratios of 0.05, 0.10, 0.15, and 0.20, respectively. Thus, it was determined that the compressive strength decreased when the EVA/cement ratio was increased.

The results of this study are similar to those of Yuanguang et al. [24], who found that compressive strength decreased by 17.5% when the EVA content was increased from 0% to 4%. Other previous studies [25,26] obtained similar results, demonstrating that the compressive strength decreased when the EVA content was increased. This low compressive strength of EVA-modified concrete (as compared to plain concrete) is likely due to microstructural damage to the cement hydrates [24,25,26]. However, in the present study, the potential causes of such strength degradation were determined to be the use of a powder-type EVA in the 3DAC material, as well as the increase in water/cement ratio over the EVA/cement ratio increase (done to secure the optimal workability obtained at a 65% flow).

#### 3.1.2. Compressive Strengths of Printed Specimens

**Figure 4:**
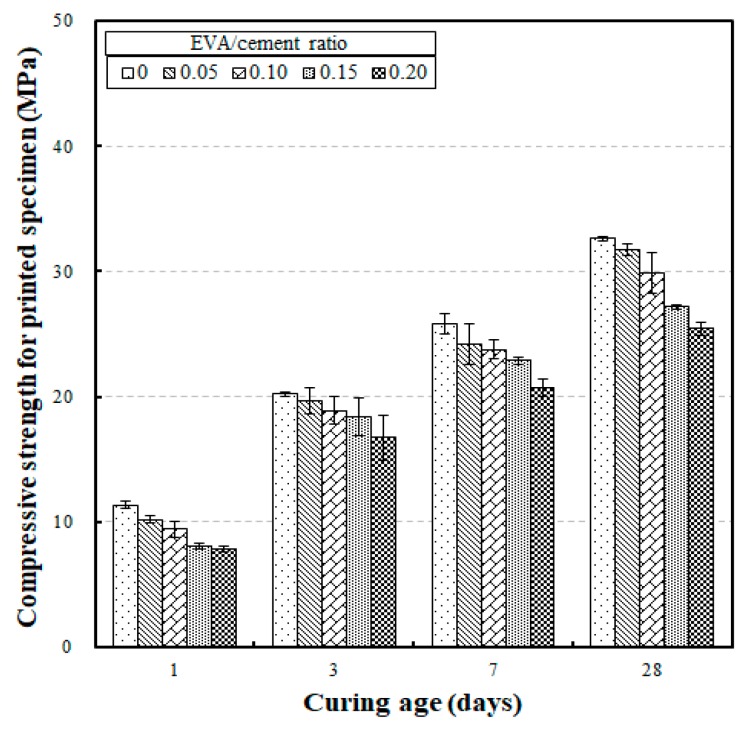
Compressive strength for printed specimens versus curing age with different EVA/cement ratios.

The compressive strength test results for printed specimens according to curing age are shown in Figure 4. The compressive strengths after curing ages of 1, 3, 7, and 28 days were 7.79 MPa to 11.34 MPa, 16.71 MPa to 20.20 MPa, 20.71 MPa to 25.79 MPa, and 25.43 MPa to 32.62 MPa, respectively. The trend in compressive strength increase with an increase in curing age was less severe than in the cast specimens. When the compressive strength of the printed specimens at a curing age of 28 days was set as the reference (i.e., 100%), the rate of compressive strength development for curing ages of one, three, and seven days were 29.7% to 34.7%, 61.9% to 67.6%, and 76.2% to 84.2%, respectively. For the cast specimens, the compressive strengths increased exponentially after a curing age of seven days. In contrast, the compressive strengths of the printed specimens increased relatively moderately after a curing age of seven days. This result may be due to an absence of compaction in the EVA-modified mortars when the printed specimens were produced.

**Figure 5:**
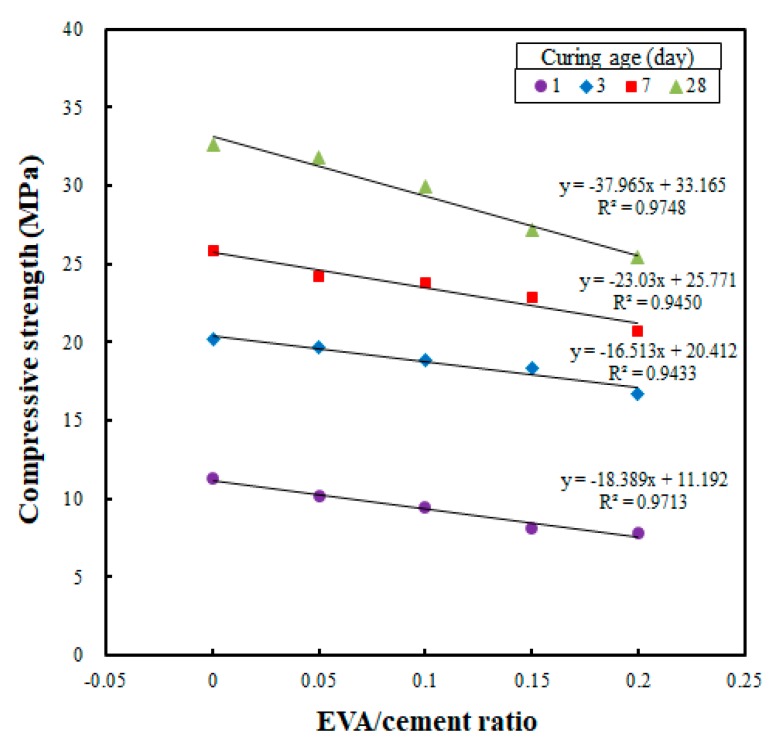
Relationship between EVA/cement ratio and compressive strength for printed specimens at different curing ages.

Figure 5 presents the results showing the correlation between the EVA/cement ratio and compressive strength of each printed specimen, allowing for an evaluation of the impact of that ratio on compressive strength development. As shown in Figure 5, the compressive strength decreased when the EVA/cement ratio was increased, as was also the case with the cast specimens. Also like the cast specimens, the slope of the linear regression indicates that the compressive strength of the printed specimen decreased rapidly when the curing age was increased. Specifically, the effect of the EVA/cement ratio on compressive strength was examined for printed specimens with a curing age of 28 days (see Figure 5). When the EVA/cement ratio of zero was set as the reference (i.e., 100%), the test results showed 97.9%, 91.6%, 83.2%, and 77.9% for the ratios of 0.05, 0.10, 0.15, and 0.20, respectively. It was observed that the compressive strength decreased as the EVA/cement ratio was increased. This was similar to what was seen in the cast specimens.

**Figure 6:**
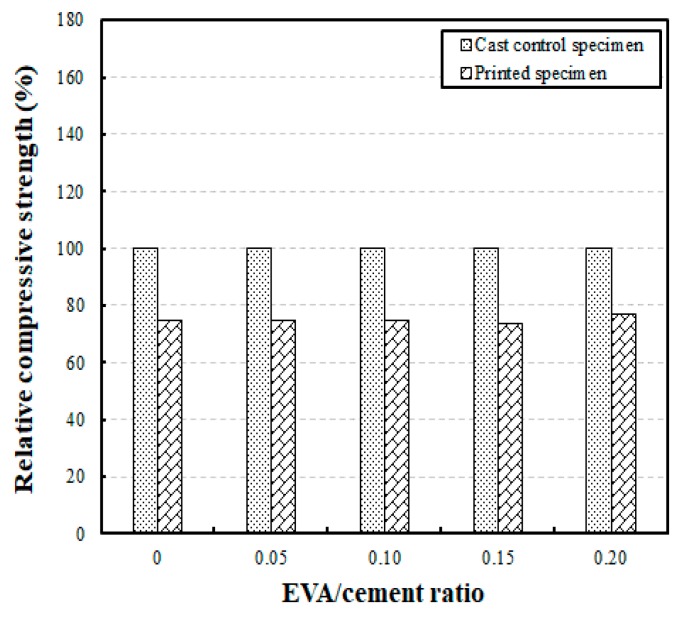
Comparison of relative compressive strengths of cast and printed specimens with different EVA/cement ratios.

A comparative analysis of the compressive strengths of the printed and cast specimens can be found in Figure 6. The results show the relative compressive strengths compared to the developed compressive strengths of the printed and cast specimens at a 28-day curing age, according to different EVA/cement ratios. The compressive strengths of the printed specimens were 74% to 77% of the compressive strengths of the cast specimens, meaning that the printed specimens’ compressive strengths decreased by 23% to 26%, relative to those of the cast specimens.

Marchment [27] compared the compressive strengths of cast and printed specimens using cement mortar, showing that the compressive strength of cast specimens at a curing age of seven days was 34 MPa, while the printed specimens at the same curing age developed compressive strengths between 8.8 MPa and 22.8 MPa. This trend in the test results is similar to what was seen in the present study. Thus, the testing of EVA-modified mortars carried out in the present work revealed that the compressive strength developed was between 32.92 MPa and 43.50 MPa in the cast specimens and 25.43 MPa and 32.62 MPa in the printed specimens, with a curing age of 28 days serving as a reference.

### 3.2. Flexural Strength

#### 3.2.1. Flexural Strengths of Cast Specimens

**Figure 7:**
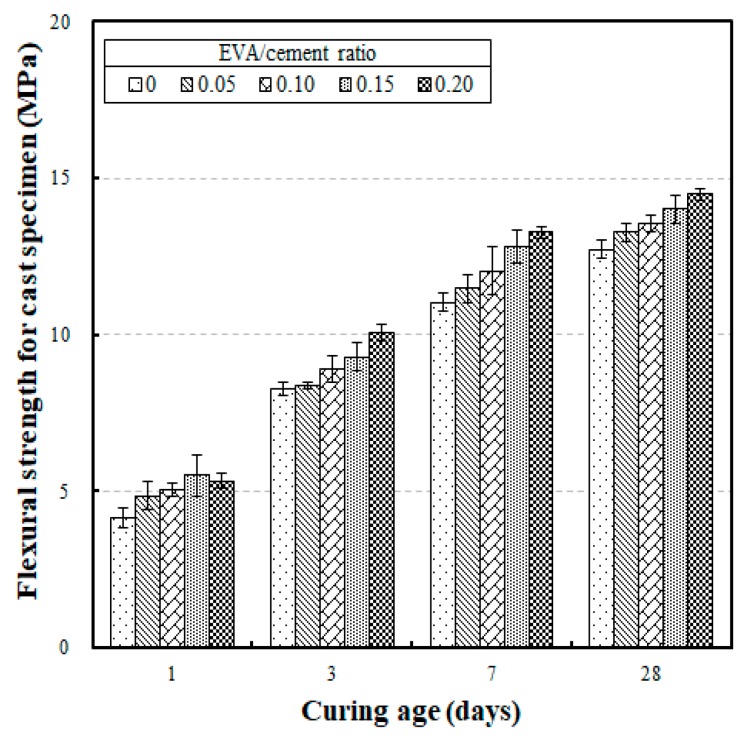
Flexural strength for cast specimens versus curing age with different EVA/cement ratios.

Figure 7 shows the flexural strength test results for the cast specimens, according to different curing ages. The results show that the flexural strengths for curing ages of 1, 3, 7, and 28 days were 4.15 MPa to 5.33 MPa, 8.26 MPa to 10.09 MPa, 11.03 MPa to 13.28 MPa, and 12.73 MPa to 14.49 MPa, respectively. The flexural strengths did not increase considerably between the curing ages of seven and 28 days. This was because the strength development at seven days already reached 86.5% to 91.6% of that which was achieved at a curing age of 28 days. This was due to the relatively high rate of flexural strength developed at an early age in the EVA-modified mortars.

**Figure 8:**
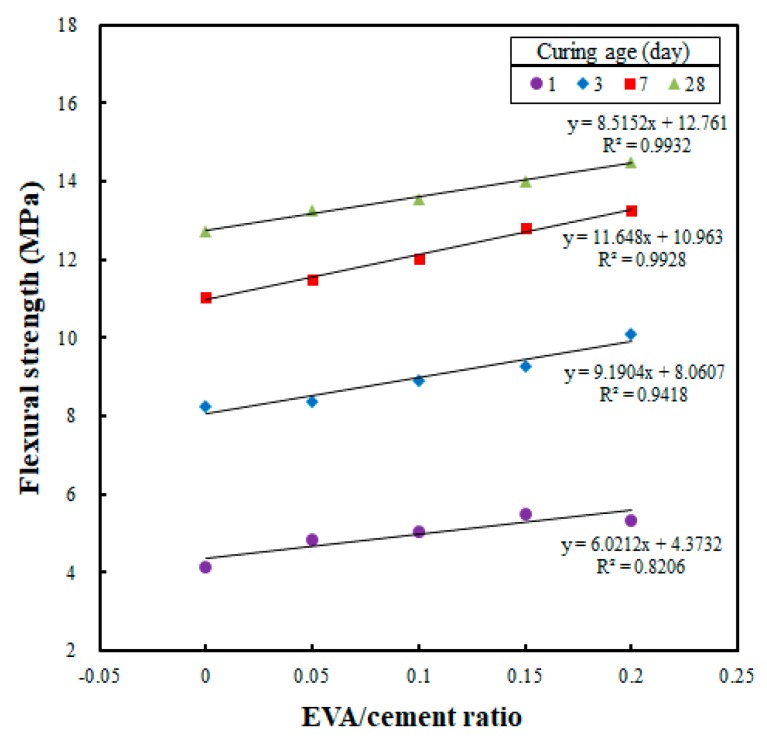
Relationship between EVA/cement ratios and flexural strengths for cast specimens at different curing ages.

Figure 8 shows the correlation between the EVA/cement ratio and flexural strength according to curing age, illustrating the effect of the EVA/cement ratio on the flexural strength of each cast specimen. This analysis revealed that the flexural strength increased as the EVA/cement ratio was increased. The linear regression slope also demonstrates that the flexural strength increased as the curing age was increased. This trend can be attributed to the EVA copolymer particles in the cement mixtures, which enhanced the adhesive strength of the cement matrix. In addition, the EVA particles absorbed the fracture energy needed to generate and propagate cracks, resulting in an increase in toughness in the cement mixtures [24].

The effect of the EVA/cement ratio on the flexural strength development of the cast specimens at a curing age of 28 days is shown in Figure 8. According to the results, when an EVA/cement ratio of zero was set as the reference (i.e., 100%), the flexural strengths of the cast specimens were 104.3%, 106.5%, 110.1%, and 113.8% when the EVA/cement ratios were 0.05, 0.10, 0.15, and 0.20, respectively. This result is similar to trends in previous studies in which the flexural strength significantly improved as the polymer content was increased. Also, the flexural strengths of the EVA-modified mortars were much better than the flexural strength of plain concrete when the EVA/cement ratio was increased [16,24,28,29].

#### 3.2.2. Flexural Strengths of Printed Specimens

**Figure 9:**
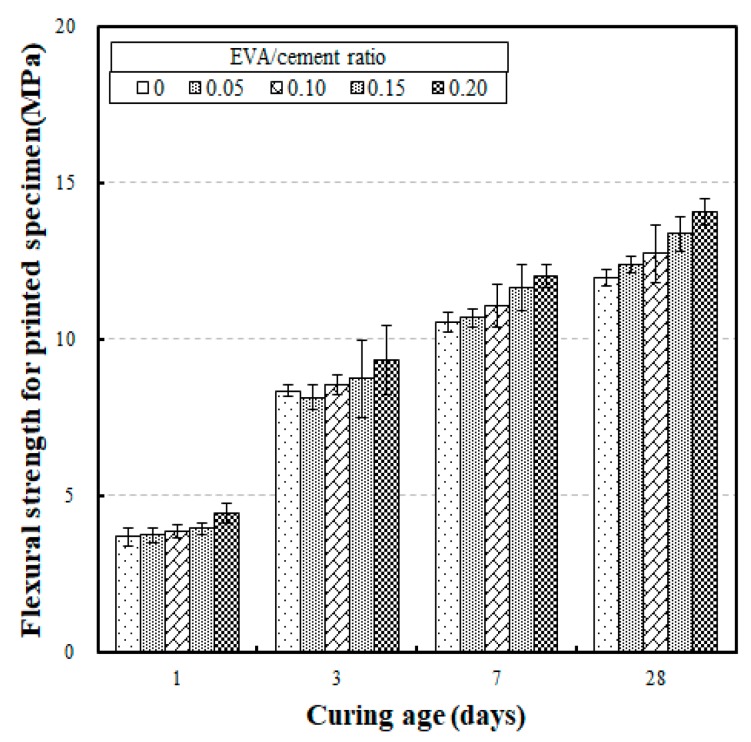
Flexural strength for printed specimens versus curing age with different EVA/cement ratios.

The flexural strength test results for printed specimens according to different curing ages are shown in Figure 9. The results show that the flexural strengths for curing ages of 1, 3, 7, and 28 days were 3.70 MPa to 4.44 MPa, 8.15 MPa to 9.35 MPa, 10.54 MPa to 12.02 MPa, and 12.73MPa to 14.49 MPa, respectively. These results are similar to the flexural strengths developed in the cast specimens discussed above. In relation to the flexural strengths at a curing age of 28 days, the flexural strengths developed at a curing age of seven days ranged between 85.4% and 88.0%. This result indicates that the rate of strength development at an early age is not significantly different from the flexural strengths of the cast specimens, even when a 3D concrete printer was used to produce them.

**Figure 10:**
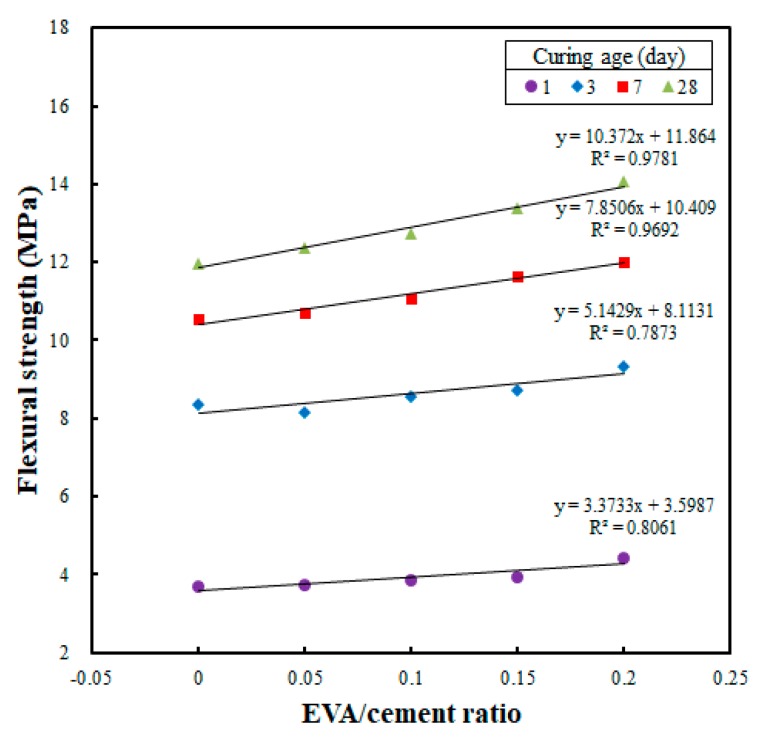
Relationship between EVA/cement ratio and flexural strength in printed specimens at different curing ages.

Figure 10 shows the correlation analysis conducted to investigate whether the flexural strength of a printed specimen would be affected by the EVA/cement ratio. The results are similar to those of the cast specimens. The flexural strength increased as the EVA/cement ratio was increased. Also, the linear regression slope demonstrates that the flexural strength increased as the curing age was increased, which is the same trend seen in the cast specimens. In addition, the flexural strength increased rapidly, from 103.4% for an EVA/cement ratio of 0.05, to 106.3% for 0.10, 111.7% for 0.15, and 117.5% for 0.20, when an EVA/cement ratio of zero was set as the reference (i.e., 100%). These results show that the rate of strength development also increased as the EVA/cement ratio was increased; the flexural strength increased to 117% at an EVA/cement ratio of 0.20. Hence, the flexural strength increased as the EVA content was increased, which is similar to what was seen in the flexural strength test of the cast specimens.

**Figure 11:**
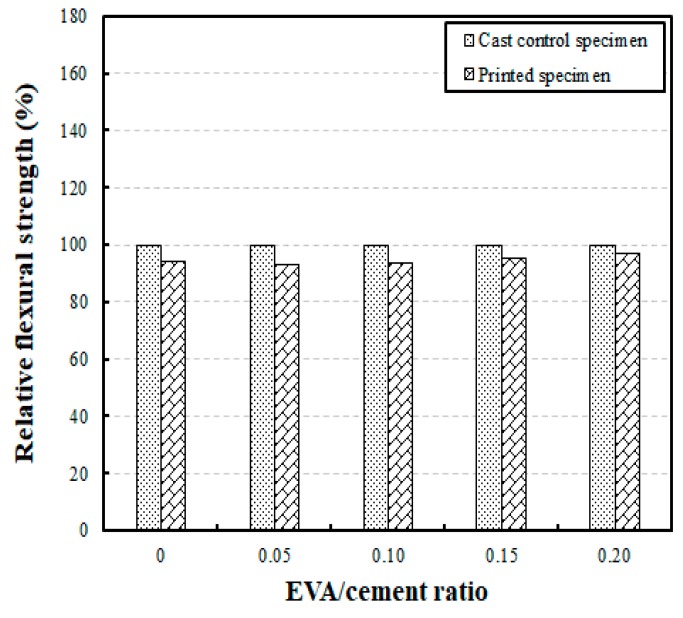
Comparison of relative flexural strengths of cast and printed specimens for different EVA/cement ratios.

Figure 11 shows the results of a comparative analysis of the flexural strengths of the printed and cast specimens. This analysis compared the flexural strengths of the printed and cast specimens at a curing age of 28 days, according to different EVA/cement ratios. The results show that the flexural strengths of the printed specimens corresponded to 93% to 97% of the cast specimens’ flexural strength, meaning that the flexural strength decreased by 3% to 7%. This was a small decrement compared to the decrement seen in the compressive strength tests. In other words, for a curing age of 28 days, the flexural strengths of the cast specimens developed up to 12.73 MPa to 14.49 MPa, and the flexural strengths of the printed specimens developed up to 11.97 MPa to 14.07 MPa. It was determined from these results that the flexural strengths of the two specimen types did not differ greatly.

## 4. Conclusions

This study experimentally investigated the compressive and flexural strengths of EVA-modified mortars for use in 3DAC applications. The compressive and flexural strengths of the cast and printed specimens were also determined and compared in order to assess the optimal properties of EVA-modified mortars for use in 3DAC applications. The following conclusions were drawn:

1. The compressive strengths at a curing age of 28 days for the cast specimens were 32.92 MPa to 43.50 MPa, and 25.43 MPa to 32.62 MPa for the printed specimens. The flexural strengths of the cast and printed specimens at a curing age of 28 days were 12.73 MPa to 14.49 MPa and 12.73 MPa to 14.49 MPa, respectively.
2. The compressive strengths of the cast and printed specimens were similar when organized according to curing age. The compressive strengths at a curing age of seven days developed from 76% to 85%, as compared to the compressive strengths after 28 days. The flexural strengths of the cast and printed specimens were also similar. The flexural strengths for seven days ranged between 85% and 92%, relative to when the curing age was 28 days.
3. If an EVA/cement ratio of zero was set as the reference (i.e., 100%), the rate of compressive strength development for the cast specimens was 75.6% when the EVA/cement ratio was 0.20; for the printed specimens, this value was 77.9%. The rate of flexural strength development for the cast specimens was 113.8% when the EVA/cement ratio was 0.20; it was 117.5% for the printed specimens.
4. Overall, the strengths of the printed specimens were lower than those of the cast specimens, by 23% to 26% and 3% to 7% for compressive and flexural strengths, respectively.

Considering these test results, the EVA-modified mortars showed high rates of strength development at early curing ages. Thus, these mortars are advantageous for 3DAC. It was also determined that the optimal EVA/cement ratios should range from 5% to 15% for 3DAC. However, the compressive and flexural strengths of the printed specimens were lower than those of the cast specimens. Moreover, the compressive strengths of the EVA-modified mortars decreased when the EVA content was increased. Therefore, these shortcomings should be examined in future research.

## Figures and Tables

**Table 1:** Properties of ordinary Portland cement.

Density (g/cm^3^)	Chemical Composition (%)			Specific Surface (cm^2^/g)
	MgO	SO_3_	Loss on Ignition	
3.14	2.91	2.41	2.16	3630

**Table 2:** Properties of silica sand.

Size (mm)	Apparent Density	Purity (%)	Water Content (%)
0.08	1.57	97.3	<0.1

**Table 3:** Properties of fly ash.

Density (g/cm^3^)	SiO_2_ (%)	Loss Ignition (%)	Specific Surface (cm^2^/g)
2.22	51.9	3.2	3651

**Table 4:** Properties of silica fume.

SiO_2_ (%)	H_2_O (%)	Loss on Ignition (%)	Bulk Density, Undensified (kg/m^3^)	Bulk Density, Densified (kg/m^3^)	Specific Surface (cm^2^/g)
96.7	<1.0	<3.0	200–350	600–700	157,700

**Table 5:** Properties of superplasticizer.

Specific Gravity (20 °C)	pH	Alkali Content (kg/m^3^)	Chloride Content (kg/m^3^)
1.05 ± 0.05	5.0 ± 2.0	0.03	0.03 × 10^−3^

**Table 6:** Properties of viscosity-modifying agent.

Appearance	pH	Bulk Density (kg/m^3^)	Moisture Content (%)	Particle Size 0.074 mm (%)
White powder	8.0–10.0	430	≤12	≥95

**Table 7:** Properties of ethylene-vinyl acetate (EVA) powder.

Solids Content (%)	Ash Content (%)	Bulk Density (kg/m^3^)	Particle Size after Redispersion (μm)	Minimum Film-Forming Temperature (°C)	Protective Colloid/Emulsifier System
98–100	9–13	470–570	0.5–8.0	4	Polyvinyl alcohol, high molecular compounds

**Table 8:** Mix proportion for the EVA-modified mortars (wt.%).

EVA/Cement Ratio	Water/Cement Ratio	Cement	Silica Sand	Fly Ash	Silica Fume	Super Plasticizer (phc*)	Viscosity-Modifying Agent (phc*)
0	0.45	28	60	8	4	(1)	(0.05)
0.05	0.46						
0.10	0.51						
0.15	0.52						
0.20	0.55						

* parts per hundred parts of cement.

## References

[1] 3D Hubs. https://www.3dhubs.com/knowledge-base/advantages-3d-printing.

[2] Dimitrove D., Schreve K., De Beer N. (2006). Advances in Three-Dimensional Printing—State of the Art and Future Perspectives. Rapid Prototyp. J..

[3] Breman B. (2012). 3-D Printing: The New Industrial Revolution. Bus. Horiz..

[4] Lam C.X.F., Mo X.M., Teoh S.H., Hutmacher D.W. (2002). Scaffold Development using 3D Printing with A Starch-Based Polymer. Mater. Sci. Eng..

[5] Wegrzyn T.F., Golding M., Archer R.H. (2012). Food Layered Manufacture: A New Process for Constructing Solid Foods. Trends. Food. Sci. Tech..

[6] Penga J. (1997). Exploratory Investigation of Solid Freeform Construction. Autom. Constr..

[7] Khoshnevis B. (2004). Automated Construction by Contour Crafting—Related Robotics and Information Technologies. Autom. Constr..

[8] Yeon K.S., Kim K.K., Yeon J., Taha M. (2018). Feasibility Study of the Use of Polymer-Modified Cement Composites as 3D Concrete Printing Material. Proceedings of the International Congress in Polymers in Concrete.

[9] ACI. https://www.concrete.org/news/newsdetail.aspx?f=51715671.

[10] Buswell B.A., Leal de Silva W.R., Jones S.Z., Dirrenberger J. (2018). 3D Printing using Concrete Extrusion: A Road Map for Research. Cem. Concr. Res..

[11] Gosselin C., Duballet R., Roux P., Gaudillière N., Dirrenberger J., Morel P.H. (2016). Large-Scale 3D Printing of Ultra-High Performance Concrete—A New Processing Route for Architects and Builders. Mater. Design..

[12] Le T.T., Austin S.A., Lim S., Buswell R.A., Law R., Gibb A.G.F., Thorpe T. (2012). Hardened Properties of High-Performance Printing Concrete. Cem. Concr. Res..

[13] Chandra S., Ohama Y. (1994). Polymers in Concrete.

[14] Wu Y.Y., Ma B.G., Wang J., Zhang F.C., Jian S.W. (2011). Study on Interface Properties of EVA-Modified Cement Mortar. Adv. Mater. Res..

[15] Mansur A.A.P., Nascimento O.L., Mansur H.S. (2009). Physico-Chemical Characterization of EVA-Modified Mortar and Porcelain Tiles Interfaces. Cem. Concr. Res..

[16] Ohama Y. (1995). Handbook of Polymer-Modified Concrete and Mortars.

[17] Hager I., Golonka A., Putanowicz R. (2016). 3D Printing of Buildings and Building Components as the Future of Sustainable Construction?. Procedia Eng..

[18] Sanjayan J.G., Nematollahi B., Sanjayan J.G., Nazari A., Nematollahi B. (2019). 3D concrete printing for construction applications. 3D Concrete Printing Technology.

[19] Nerella V.N., Mechtcherine V., Sanjayan J.G., Nazari A., Nematollahi B. (2019). Studying the printability of fresh concrete for formwork-free concrete onsite 3D printing technology (CONPrint3D). 3D Concrete Printing Technology.

[20] Yeon K., Kim K.K., Yeon J., Lee H.J. (2019). Fresh Properties of EVA-Modified Cementitious Mixtures for Use in Additive Construction via Extrusion. Materials.

[21] ASTM C348-14. https://www.astm.org/DATABASE.CART/HISTORICAL/C348-14.htm.

[22] ASTM C109/C109M-02. https://www.astm.org/DATABASE.CART/HISTORICAL/C109C109M-02.htm.

[23] ASTM C349-18. https://www.astm.org/Standards/C349.

[24] Yuanguang Y., Bin Y., Qunliang S., Xin T., Xie Y. (2015). Mechanical Properties of EVA-Modified Cement for Underground Gas Storage. J. Nat. Gas Sci. Eng..

[25] Medeiros M.H.F., Helene P., Selmo S. (2009). Influence of EVA and Acrylate Polymers on Some Mechanical Properties of Cementitious Repair Mortars. Constr. Build. Mater..

[26] Liu S., Kong Y., Wan T., Zhao G. (2018). Effects of Thermal-Cooling Cycling Curing on the Mechanical Properties of EVA-Modified Concrete. Constr. Build. Mater..

[27] Marchment T., Sanjayan J.G., Nematollahi B., Xia M., Sanjayan J.G., Nazari A., Nematollahi B. (2019). Interlayer Strength of 3D Printed Concrete: Influencing Factors and Method of Enhancing. 3D Concrete Printing Technology.

[28] Weng T.L., Lin W.T., Li C.H. (2017). Properties Evaluation of Repair Mortars Containing EVA and VA/VeoVa Polymer Powders. Polym Polym. Compos..

[29] Ahmed S.F.U. (2011). Mechanical and Durability Properties of Mortars Modified with Combined Polymer and Supplementary Cementitious Materials. J. Mater. Civ. Eng..

